# A universal precautions approach to reducing stigma in health care: getting beyond HIV-specific stigma

**DOI:** 10.1186/s12954-022-00658-w

**Published:** 2022-07-07

**Authors:** Carla Treloar, Elena Cama, Kari Lancaster, Loren Brener, Timothy R. Broady, Aaron Cogle, Darryl O’Donnell

**Affiliations:** 1 Centre for Social Research in Health, UNSW Sydney, Sydney, NSW 2052 Australia; 2 National Association of People With HIV Australia (NAPWHA), Sydney, Australia; 3 Australian Federation of AIDS Organisations (AFAO), Sydney, Australia

**Keywords:** Stigma, HIV, Universal precautions, Equity

## Abstract

**Background:**

Delivery of effective health care is hampered by stigma, the social processes that attach negative judgement and response to some attributes, conditions, practices and identities. Experiencing or anticipating stigma can lead to a range of practical impacts, including avoidance of health care. While we are concerned about the stigma that is attached to HIV, this commentary makes the argument that the health system is burdened by stigma of many origins.

**Main body:**

Reducing stigma is a key issue in improving quality of health care. Our focus on HIV is about providing better care in a non-judgemental, respectful and dignified manner which enhances the health and well-being of individuals as well as delivering benefit to society at large through better population health outcomes. However, the same could be said for the numerous possible attributes, conditions, practices and identities that attract stigma. It is unrealistic to expect health systems to respond to siloed appeals for change and action. A unifying logic is needed to propel concerns about stigma to the front of the queue for action by health systems.

**Conclusion:**

This commentary suggests the need for a universal precautions approach to stigma in health care, that focuses on recognising that all people may experience stigma and discrimination targeted at one or more aspects of their identities, attributes, practices and health conditions. Drawing on health system precepts of equity, access and quality of care, we argue that a universal precautions approach to reducing stigma of all origins can effect everyday aspects of policy, procedure and practice to improve outcomes for individuals and for population health.

## Background

There are several different approaches to understanding stigma and its relationship to health. Goffman [1] proposed that stigma occurs when an individual’s or group’s identity is perceived to be “spoiled” or flawed, and subsequently the person or group is shunned by the broader social group. A well-used definition poses stigma as process consisting of the co-occurrence of labelling, stereotyping, separation, status loss and discrimination together in a situation where social, political and economic power is exercised [2]. Weiss and colleagues [3] build on this to suggest that health-related stigma is predicated on a perceived health problem or condition (such as HIV), or other feature of identity associated with the health condition (such as practices associated with HIV transmission). Stigma associated with a health condition can intersect with negative social judgements related to features of identity that are not associated with the health condition and with other major classes of structural disadvantage (gender, race, class, for example) and cumulatively contribute to poor health outcomes [4, 5].

Jones and colleagues provided an outline of six dimensions on which attributes, conditions, practices and identities that may be the target of stigma are expected to vary in a social process: concealability (the extent of visibility to others), course (the extent of persistence over time), disruptiveness (the extent of interference with smooth social interactions), aesthetics (the potential to evoke a disgust reaction), origin (whether present at birth, accidental or deliberate) and peril (the extent of perceived personal threat or potential for contagion) [6]. Socio-ecological models have been used to define interacting, inter-connected levels of stigma and its manifestations: public policy and structural levels (national and local laws and policies), organisational (organisations, social institutions, workplaces), interpersonal (family, friends, social networks) and individual (knowledge, attitudes, skills) [7–9].

Following Goffman’s [1] understanding, stigma limits participation, opportunity and social acceptance through discrediting and “othering”, enabling the enactment of discrimination. Stigma has been noted as a major influence on population health and a fundamental cause of health inequities, operating through multiple mechanisms, which disrupt or inhibit “access to multiple resources—structural, interpersonal, and psychological—that could otherwise be used to avoid or minimize poor health” [10] (p. 819). Previous experience of stigma (one’s own or vicariously) or anticipation of stigma can mean individuals are reluctant to attend health services for prevention, care and treatment. When enacted in health settings, stigma can result in extended waiting times, provision of sub-standard care, inappropriate and excessive use of hygiene and infection control procedures, denial of care, and physical or verbal abuse [11].

In HIV, and other infectious diseases, the notion of peril or contagion differentiates these identities from other health (such as mental health or obesity) or social conditions (such as illiteracy or poverty); however, concerns about stigma in each example will result in avoidance of health care. In terms of engaging with the health system, issues of concealability, or passing, are very pertinent. Situations can give rise to stigma when particular attributes, conditions, practices, or identities are made more visible or perceptible to others, and by association, through for example, attendance at specific health services or places, the presence of material objects, or the time it takes to access care. A person attending a specific health care service (e.g. sexual health service, or needle and syringe programme), materials associated with care (e.g. medication packaging, or preventative technologies such as condoms and sterile injecting equipment), or indicators of time use (e.g. absence from work or family activities for health care) can be associated with stigmatised attributes, practices and identities. These aspects can act as symbols to the outside world of an identity to be shunned but can also function as reminders to individuals, as internalised stigma, of the contingent social and cultural meanings attached to these places, times and things.

For HIV, research shows that each step of the care cascade is negatively impacted by stigma, resulting in significant cumulative impact on health outcomes by limiting connection to and engagement with testing, diagnosis and treatment [9]. For example, a recent meta-analysis showed that people who had experienced HIV-related stigma were 21% less likely to attend health and social services and 32% less likely to adhere to treatment than those who did not experience stigma [12]. Our research has shown direct implications of HIV stigma for reduced screening, diagnosis and treatment uptake. For example, we have shown that experiences of stigma and discrimination are associated with reduced willingness to disclose an HIV-positive status [13] and reduced HIV treatment uptake [14, 15], and that the negative judgements towards HIV and subsequent health impacts can extend to HIV-negative men who have sex with men [16]. These results indicate the need for health workers to understand the flow-on effects of stigma experienced in their services to other settings and encounters, and the hesitancy people may have in coming to their service after experiencing stigma elsewhere. Analyses to examine the economic impacts of these effects of stigma are nascent. However, in a modelling study from South Africa, 35–50% of infections among newborns of women with HIV were attributed to the cumulative effect of stigma at each step of the care cascade [17] with subsequent individual and societal costs.

Beyond HIV, the range of conditions which attract stigma is very broad. Pachankis and colleagues’ [18] work in categorising attributes, conditions, practices and identities that may be the target of stigma is illustrative of the multiple, complex and intersecting nature of stigma. Their work categorised 93 identities, conditions and attributes reported as attracting stigma. In a sample of 1,025 adults in the USA, most participants (> 95%) indicated that they lived with at least one attribute that could potentially give rise to stigma, and 90% reported more than one, with an average of six. Indeed, the authors argue that “stigma affects a substantial segment of the U.S. population at any given time, with most individuals being stigmatised at some point in their lives” (p451). There exists much potential for people to experience stigma, given the range of attributes, conditions, practices and identities to which it can attach and the range of contexts and settings in which can heighten the experience of stigma. As a result, we cannot know which (or which combination of) statuses will be foremost in an individual’s minds when they seek or provide health care or the conditions of health care settings that can enable or reduce the possible manifestations of stigma.

### Main text: universal precautions approach to stigma

In pushing forward science and policy to effectively reduce stigma and ameliorate conditions that allow for the manifestation of discriminatory practices, we look to the growing literature on the impact of quality of care. The Lancet Global Health Commission on High Quality Health Systems highlighted the excessive costs, morbidity and mortality associated with poor-quality care around the world [19]. The Commission also emphasised the inequitable distribution of poorer quality care and association with stigma: “Quality of care is worst for vulnerable groups, including the poor, the less educated, adolescents, those with stigmatised conditions, and those at the edges of health systems, such as people in prisons” (p e1196). While it is not only stigma that can detract from quality care and produce inequitable health outcomes, previous authors have called for “strategically colluding with and leveraging” (p 864) accepted principles of quality improvement [20] to generate buy-in from health system administrators for stigma reduction efforts.

Likewise, we are arguing for a strategic collusion between the equity pillar of health care quality, stigma reduction and another precept of health care: universal precautions. Universal precautions have origins in infectious disease, specifically HIV [21]. These precautions cover a range of practices designed to be consistently applied as everyday procedures in all encounters with patients regardless of HIV status to prevent exposure to blood and body fluids. The ability to enact universal precautions at the point of delivery of care is predicated on an enabling environment that provides supportive policies, time and training for health workers to enact universal precautions, and for these practices to become integrated into the everyday, not exceptionally applied. The use of universal precautions has also been used as a stigma reduction intervention for HIV [22]: that is, if you treat every situation the same in terms of the potential for infection, then the particularities of an individual are decentred as a source of risk or contagion concern, and hence the conditions and assumptions that give rise to stigma might also be shifted. The appropriation of the universal precautions principle has been done in other areas, such as efforts to promote health literacy [23]: “Health care providers taking universal precautions assume that all patients may have difficulty comprehending health information and accessing health services” [24] (page e217).

In this commentary, we are arguing for a different appropriation of the well-understood notion of universal precautions. We suggest that a universal precautions approach can be used for stigma reduction not towards any one attribute, condition, practice or identity, but towards a much larger enemy of effective health care: stigma, of all types and origins. A universal precautions approach to stigma (and quality of care) assumes that all patients are fearful of exclusion or poor treatment on the basis of one or more attributes, conditions, practices or identities, that multiple settings and situations might give rise to stigma and discrimination (whether intended or not), and that this fundamentally undermines provision of effective health care.

This approach recognises the strains on a health system being asked to do innumerable things. While we are focused on the corrosive effects of HIV stigma on quality of care, other groups will be advocating for action to reduce the harms associated with a broad range of other stigmatised attributes, conditions, practices and identities to increase access to and quality of health services. Expecting the health system, or other institutions of social responsibility, to respond with equal and unique attention to each of our very important agendas is impractical and unrealistic. Further, it is impractical to ask front-line service providers or those drafting policies and procedures to disentangle the likely complex web of experiences and concerns that individuals and services will need to address. Because stigma threatens to undermine the effectiveness of every step of the care pathway for every condition that the health system seeks to address, we need a unifying logic and approach to practice that can propel concerns about stigma to the front of the queue for action by health systems.

The call for stigma reduction interventions that tackle multiple and intersecting dimensions of stigma at once is not new [11, 25]. Although there is little specific guidance on how to do this, some principles from the existing literature can be applied. Developing a universal precautions approach to stigma reduction needs to position the expertise and knowledge of people with lived experience in the centre of these efforts, as has been defined as best practice [11]. A universal precautions approach can activate all levels of the social–ecological model as a cross-health system response, especially interrogation of organisational policies and procedures that actively or by default exclude or alienate specific people or groups. Further than this, and because of the multi-level production of stigma, we need a way to frame this not just as a problem or responsibility of health worker attitude or behaviour. The ways in which stigma is (re)produced and made possible in a range of practices, and the stigmatising potential of policies, time, place, materials, institutions, media and law [26] should be considered within this framework.

The specific aspects of a universal precautions approach to stigma reduction require development work. We suggest that some principles to pursue in this approach would include generating system-wide understanding (from funders to front-line workers) of the pervasive experience of stigma in people’s lives and the impact of stigma on the social and economic project of health care. This approach could focus on undoing assumptions that some attributes, conditions, practices and identities are inherently stigma-attracting. Instead, this approach could emphasise the choices made in current policy, procedure and practice to differentiate these attributes, conditions, practices and identities and ascribe negative meaning and response to them [27]. What could be most challenging is balancing a universal approach with the need for specificities regarding some attributes, conditions, practices and identities [11]. For example, where there is criminalisation of some aspects related to the HIV response (homosexuality in some countries; drug use in just about all countries; transmission of HIV in some contexts), this will require specific attention over and above general work in promoting a non-stigmatising health care system, as well as understanding and responding to important specifics (or categories of specificity) of stigma associated with other health conditions.

Building a stigma universal precautions framework provides us the opportunity to embed the principle that responsibility for implementation, evaluation and accountability of stigma reduction efforts is shared, and relates to practices in the every day. The stigma reduction literature emphasises the over-reliance on interventions that are focused on interpersonal or individual levels using educational or contact interventions, with few interventions that tackle organisational or structural levels [28]. Techniques such as quality improvement approaches [29] and developing measures of structural stigma [30] provide guidance for how to build a stigma universal precautions framework that spans the levels of the ecological model in design, implementation and evaluation. We will be taking up this challenge in an upcoming project in the Australian health system, where we will have opportunity to develop this framework and then build and test strategies to put this framework into practice.

## Conclusions

The costs of stigma on the effectiveness of health systems are too great to ignore. But stigma is pervasive in the human experience and attached to numerous experiences of life. Health systems cannot tackle separate stigma reduction programmes for the myriad identities, conditions, attributes and practices, or even a tenth of these: how would we choose which (or which combinations) are more or less worthy? A universal precautions approach to stigma reduction is aligned with and can leverage health system precepts of equity, access and quality. To operationalise this, we need programs for stigma reduction which are built into everyday practices and which educate all on the corrosive effects of stigma on the overall goals of health systems; how not addressing stigma damages the lives of the majority of the population, makes everyone’s job harder, and costs our society. We need high-quality science that tackles multiple and intersecting forms of stigma while being attentive to specificities that make a difference. We need to develop systems of accountability that map onto all levels of the ecological model. And, finally, we need to ensure that people with lived experience are centrally involved in each step of this work.

## Data Availability

Not applicable for this perspective submission.

## References

[1] Goffman E (1963). Stigma: notes on the management of spoiled identity.

[2] Link B, Phelan J (2001). Conceptualizing stigma. Ann Rev Sociol.

[3] Weiss M, Ramakrishna J, Somma D (2006). Health-related stigma: Rethinking concepts and interventions. Psychol Health Med.

[4] Turan JM, Elafros MA, Logie CH, Banik S, Turan B, Crockett KB (2019). Challenges and opportunities in examining and addressing intersectional stigma and health. BMC Med.

[5] Henkel KE, Brown K, Kalichman SJS, Compass PP (2008). AIDs related stigma in individuals with other stigmatized identities in the USA: a review of layered stigmas. Soc Personal Psychol Compass.

[6] Jones E, Farina A, Hastorf A, Markus H, Miller D, Scott R. Social stigma: The psychology of marked relationships. New York: W. H. Freeman; 1984.: W. H. Freeman; 1984.

[7] Cook J, Purdie-Vaughns V, Meyer I, Busch J (2014). Intervening within and across levels: a multilevel approach to stigma and public health. Soc Sci Med.

[8] Stangl AL, Earnshaw VA, Logie CH, van Brakel W, C Simbayi L, Barré I (2019). The health stigma and discrimination framework: a global, crosscutting framework to inform research, intervention development, and policy on health-related stigmas. BMC Med.

[9] Nyblade L, Mingkwan P, Stockton MA (2021). Stigma reduction: an essential ingredient to ending AIDS by 2030. The Lancet HIV.

[10] Hatzenbuehler M, Phelan J, Link B (2013). Stigma as a fundamental cause of population health inequalities. Am J Public Health.

[11] Nyblade L, Stockton MA, Giger K, Bond V, Ekstrand ML, Lean RM (2019). Stigma in health facilities: why it matters and how we can change it. BMC Med.

[12] Rueda S, Mitra S, Chen S, Gogolishvili D, Globerman J, Chambers L (2016). Examining the associations between HIV-related stigma and health outcomes in people living with HIV/AIDS: a series of meta-analyses. BMJ Open.

[13] Cama E, Brener L, Slavin S, de Wit J (2020). The relationship between negative responses to HIV status disclosure and psychosocial outcomes among people living with HIV. J Health Psychol.

[14] Cama E, Brener L, Slavin S, de Wit J (2015). The impact of HIV treatment-related stigma on uptake of antiretroviral therapy. AIDS Care.

[15] Newman CE, Mao L, Persson A, Holt M, Slavin S, Kidd MR (2015). 'Not until I'm absolutely half-dead and have to:' accounting for non-use of antiretroviral therapy in semi-structured interviews with people living with HIV in Australia. AIDS Patient Care STDS.

[16] Broady TR, Brener L, Hopwood M, Cama E, Treloar C, Holt M (2020). HIV stigma by association among Australian gay and bisexual men. AIDS.

[17] Prudden HJ, Hamilton M, Foss AM, Adams ND, Stockton M, Black V (2017). Can mother-to-child transmission of HIV be eliminated without addressing the issue of stigma? Modeling the case for a setting in South Africa. PLoS ONE.

[18] Pachankis JE, Hatzenbuehler ML, Wang K, Burton CL, Crawford FW, Phelan JC (2018). The burden of stigma on health and well-being: a taxonomy of concealment, course, disruptiveness, aesthetics, origin, and peril across 93 stigmas. Pers Soc Psychol Bull.

[19] Kruk ME, Gage AD, Arsenault C, Jordan K, Leslie HH, Roder-DeWan S (2018). High-quality health systems in the sustainable development goals era: time for a revolution. Lancet Glob Health.

[20] Knaak S, Patten S, Ungar T (2015). Mental illness stigma as a quality-of-care problem. Lancet Psychiatr.

[21] Centers for Disease Control (1988). Perspectives in disease prevention and health promotion update: Universal precautions for prevention of transmission of human immunodeficiency virus, hepatitis B virus, and other bloodborne pathogens in health-care settings. Morb Mortal Wkly Rep.

[22] Feyissa GT, Lockwood C, Woldie M, Munn Z (2019). Reducing HIV-related stigma and discrimination in healthcare settings: a systematic review of quantitative evidence. PLoS ONE.

[23] Brown DR, Ludwig R, Buck GA, Durham D, Shumard T, Graham SS (2004). Health literacy: universal precautions needed. J Allied Health.

[24] Liang L, Brach C (2017). Health literacy universal precautions are still a distant dream: analysis of U.S.. Data Health Lit Pract.

[25] van Brakel WH, Cataldo J, Grover S, Kohrt BA, Nyblade L, Stockton M (2019). Out of the silos: identifying cross-cutting features of health-related stigma to advance measurement and intervention. BMC Med.

[26] Seear K, Lancaster K, Ritter A (2017). A new framework for evaluating the potential for drug law to produce stigma: insights from an australian study. J Law Med Ethics.

[27] Paterson B, Hirsch G, Andres K (2013). Structural factors that promote stigmatization of drug users with hepatitis C in hospital emergency departments. Int J Drug Policy.

[28] Rao D, Elshafei A, Nguyen M, Hatzenbuehler ML, Frey S, Go VF (2019). A systematic review of multi-level stigma interventions: state of the science and future directions. BMC Med.

[29] Ikeda DJ, Nyblade L, Srithanaviboonchai K, Agins BD (2019). A quality improvement approach to the reduction of HIV-related stigma and discrimination in healthcare settings. BMJ Glob Health.

[30] Unger T. Design Prototypes for Measuring Structural Stigma in Health-Care Settings. Ottawa, Canada: Mental Health Commission of Canada; 2021.

